# Investigation on the Carrier Dynamics in P-I-N Type Photovoltaic Devices with Different Step-Gradient Distribution of Indium Content in the Intrinsic Region

**DOI:** 10.3390/mi16070833

**Published:** 2025-07-21

**Authors:** Yifan Song, Wei Liu, Junjie Gao, Di Wang, Chengrui Yan, Bohan Shi, Linyuan Zhang, Xinnan Zhao, Zeyu Liu

**Affiliations:** School of Microelectronics, Northwestern Polytechnical University, Xi’an 710072, China; syf1274639733@mail.nwpu.edu.cn (Y.S.);

**Keywords:** InGaN solar cells, carrier dynamics, step-gradient distribution, in content

## Abstract

InGaN-based photovoltaic devices have attracted great attention due to their remarkable theoretical potential for high efficiency. In this paper, the influence of different distributions of step-gradient indium content within the intrinsic region on the photovoltaic performance of P-I-N type InGaN/GaN solar cells is numerically investigated. Through the comprehensive analysis of carrier dynamics, it is found that for the device with the indium content decreasing stepwise from 50% at the top to 10% at the bottom in intrinsic region, the photovoltaic conversion efficiency is increased to 10.29%, which can be attributed to joint influence of enhanced photon absorption, reduced recombination rate, and optimized carrier transport process.

## 1. Introduction

InGaN alloys are studied increasingly as a prospective material for photovoltaic applications in harsh environments due to their high thermal stability and excellent radiation resistance [1,2,3,4,5]. Moreover, InGaN alloys with a tunable direct bandgap (spanning from 0.7 eV to 3.4 eV), a high absorption coefficient (>10^5^ cm^−1^ at the band edge), and a high carrier mobility make it possible to achieve high-performance solar cells [6,7,8,9]. A photovoltaic conversion efficiency (PCE) of 50% has been predicted for InGaN-based solar cells [10].

However, the PCE of experimentally fabricated single-junction InGaN solar cells achieved so far remains less than 5%, which is significantly lower than the theoretical prediction [11,12,13]. The performance of conventional P-I-N type GaN/InGaN/GaN solar cells with a sandwich structure strongly relies on the properties of the low-doped InGaN layer acting as the light absorption region [14,15,16]. To improve the PCE of InGaN solar cells, a higher indium (In) content of InGaN is required to push the operating wavelength towards longer wavelengths in the spectral region to match a larger distribution of the solar spectrum [10]. Nevertheless, it is well known that for the high In content InGaN solar cells, the lattice mismatch and phase separation are exacerbated, leading to the formation of dislocations during epitaxial growth [17,18]. These dislocations, acting as nonradiative recombination centers, impede carrier transport and reduce carrier collection efficiency, ultimately degrading the photovoltaic performance of InGaN solar cells [19,20].

In parallel with significant efforts to enhance the film quality, it is cost-effective to improve the PCE of InGaN solar cells through the design of device structures. The proper design of the distribution of In content in the intrinsic region (I-region) is regarded as one of the most effective approaches to enhance device performance [21,22,23,24]. For example, the step-graded interlayers are introduced between GaN and InGaN layers to reduce the energy barrier height and polarization effects caused by high In content, thereby significantly improving the PCE to 3.08% [22]. In addition, the interlayer film with a properly designed In content distribution is inserted into the active region of the InGaN solar cell, which significantly reduces the Shockley-Read-Hall (SRH) recombination [23]. Lately, an In_x_Ga_1−x_N/GaN superlattice structure with a stepwise graded distribution of In content has been introduced into the active region of InGaN solar cells, which can optimize carrier transport and significantly enhance the PCE of high In content solar cells to 22.6% in numerical simulations [24]. Despite these promising results, the carrier dynamics of the step-gradient distribution In content have not yet been investigated.

Therefore, in our work, three samples with different step-gradient distributions of In content are designed based on the conventional P-I-N type InGaN solar cell structure. It shows that the proper design of the distribution of In content within the I-region can effectively enhance the photon absorption, reduce carrier recombination, and facilitate the carrier transport, simultaneously, thereby improving the photovoltaic performance of InGaN solar cells. Furthermore, the PCE of conventionally reported P-I-N InGaN solar cells has been reported to be below 5%, which is lower than the 10.29% achieved in this work [22,23].

## 2. Sample Structure and Simulation Parameters

**Figure 1:**
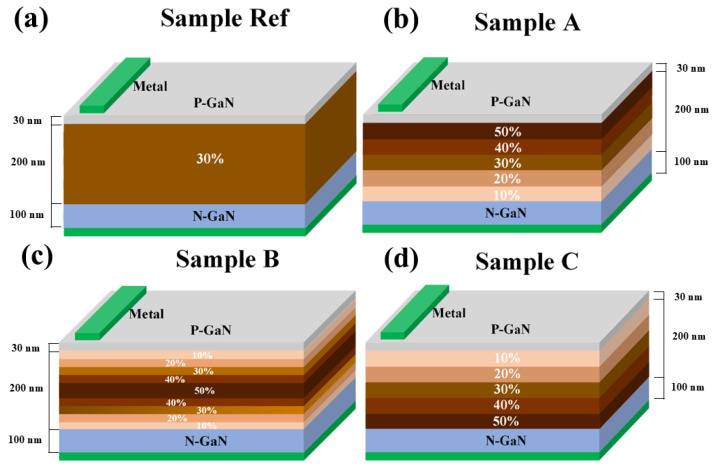
Schematic structure of sample (**a**) Ref, (**b**) A, (**c**) B, and (**d**) C.

The structures of the InGaN solar cells are illustrated in Figure 1. In all samples, the device area is 10 × 10 μm^2^ and the total thickness is 0.33 μm. The I-region consists of an InGaN layer with a thickness of 200 nm, while both the P-region and N-region are composed of GaN. The thicknesses of the P-region and N-region are 30 nm and 100 nm, respectively, and both regions are doped at a concentration of 1 × 10^18^ cm^−3^. As shown in Figure 1, for sample Ref, a uniform In content of 30% is distributed over the entire I-region. For sample A, the In content in the I-region is decreased from 50% at the top to 10% at the bottom. For sample B, the In content in the I-region increased from 10% at the top to 50% and then decreased to 10% at the bottom. In contrast to sample A, for sample C, the In content in the I-region is increased from 10% at the top to 50% at the bottom. The average In content $\bar{x}$ in the I-region is calculated using the following formula:

(1) $$\bar{x}=\frac{1}{n}\sum_{i=1}^{n}x_{i}=\frac{x_{1}+x_{2}+\cdots +x_{n}}{n}$$

where *x_i_* is defined as the In content of each individual step layer, and *n* is the total number of steps. According to Equation (1), the average In content in the I-region is the same for all samples. In short, the structures of all samples are identical, except for the distribution of In content in the I-region.

In this work, the Silvaco TCAD software (Atlas-5.26.1.R) is employed to simulate all devices. All simulations are conducted under a temperature of 300 K and AM 1.5 G standard solar illumination. It is well established that incident light on a solar cell surface undergoes a trapping process involving reflection and optical losses [25]. However, this work focuses on the impact of the step-gradient distribution of In content in the active region on device performance. To simplify the analysis, it is assumed that 100% of incident photons can be absorbed by the solar cells in our simulations. During the simulation, the SRH recombination model, carrier drift-diffusion model, low-field mobility model, absorption coefficient model, Adachi model, and polarization model are utilized [26]. Additionally, the SRH model is described by the following equation [27]:

(2) $$R_{srh}\left(cm^{-3}s^{-1}\right)=\frac{np-n_{i}^{2}}{\tau_{p}^{srh}\left[n+n_{i}.\exp\left(\frac{E_{trap}-E_{i}}{kT}\right)\right]+\tau_{n}^{srh}\left[p+n_{i}.\exp\left(\frac{E_{i}-E_{trap}}{kT}\right)\right]}$$

where *n* and *p* denote the electron and hole concentrations, *T* is the temperature in Kelvin, $E_{i}$ is the intrinsic Fermi level, $n_{i}$ is the intrinsic carrier concentration, *k* is the Boltzmann constant, $\tau_{p}^{\mathrm{srh}}$ and $\tau_{n}^{\mathrm{srh}}$ denote the minority carrier lifetime, and $E_{trap}$ is the level of impurities.

The carrier drift-diffusion transport equation and the low field mobility equation are as follows [27]:

(3) $$\;\;\vec{J}=q\mu_{p}\left(p\vec{E}-\frac{Tk}{q}\frac{dp}{dx}\right)+q\mu_{n}\left(n\vec{E}+\frac{Tk}{q}\frac{dn}{dx}\right)$$

(4) $$\mu_{i}=\mu_{\min,i}+\frac{\mu_{\max,i}-\mu_{\min,i}}{1+{\left(N/N_{g.i}\right)}^{r_{i}}}$$

where $\mu_{p}$ and $\mu_{n}$ are the mobilities of holes and electrons, respectively, *K_0_* is Boltzmann’s constant, $\mu_{max,i}$ is the mobility of the undoped material, $\mu_{min,i}$ represents the mobility in the highly doped material, $N_{g,i}$ is the doping concentration at which the mobility was ($\mu_{max,i}$ − $\mu_{min,i}$)/2, and $r_{i}$ is a measure of how quickly the mobility changed from $\mu_{min,i}$ to $\mu_{max,i}$, and *N* is the doping concentration.

Furthermore, the absorption coefficient and the optical refractive index parameter are calculated using the following equation [27]:

(5) $$\alpha =10^{5}\sqrt{a\left(hv-E_{g}\right)+b{\left(hv-E_{g}\right)}^{2}}{\mathrm{cm}}^{-1}$$

(6) $$n^{2}\left(v\right)=A{\left(\frac{hv}{E_{g}}\right)}^{-2}\left\{2-\sqrt{1+\left(\frac{hv}{E_{g}}\right)}-\sqrt{1-\left(\frac{hv}{E_{g}}\right)}\right\}+B$$

where *h* is Planck’s constant, *v* is the photon frequency, and *a* and *b* are dimensionless fitting parameters.

There are significant polarization effects in GaN devices, which are described by the following equation [28]:

(7) $$\left\{\begin{array}{c} P_{t}=P_{sp}+P_{pi}\\ P_{pi}=2\frac{a_{s}-a_{0}}{a_{0}}\left(E_{31}-\frac{C_{13}}{C_{33}}E_{33}\right) \end{array}\right.$$

where *P_t_* is the total polarization, *P_sp_* and *P_pi_* are the spontaneous polarization and piezoelectric polarization, respectively, *E*_31_ and *E*_33_ are the piezoelectric constants, and *C*_13_ and *C*_33_ are the elastic constants. *a*_0_ is the lattice constant of the material layer, and *a_S_* is the average value of the lattice constant of the layers directly above and below this layer. The specific values of the parameters used in the simulation are shown in Table S1 of the Supplementary Information.

## 3. Results and Discussion

Considering the model mentioned in Section 2 of the article, the simulated photovoltaic performance parameters, including open-circuit voltage (Voc), short-circuit current density (Jsc), fill factor (FF), and photovoltaic conversion efficiency (PCE), are calculated and summarized in Table 1. The parameters of sample Ref are almost identical to those reported in the literature for InGaN solar cells with similar structures, thus confirming the reliability of our simulation results [29,30]. Despite the average In content of 30% in the I-region being the same for all samples, the device performance differs significantly among the four samples. Notably, the PCEs of samples A, B, and C, which are configured with a step-gradient In content distribution in the I-region, are all higher than that of sample Ref with a traditional uniform distribution of In content. Particularly, the PCEs of samples A and B exhibit substantial enhancements, being 127.62 and 113.75 times greater than that of sample Ref, respectively. According to the analysis of Voc, Jsc, and FF values in Table 1, the PCE differences among the four samples are primarily caused by the variations in Jsc. For example, the Jsc of sample Ref is 0.05 mA/cm^2^, while those of samples A and B are significantly higher, at 10.35 mA/cm^2^ and 9.60 mA/cm^2^, respectively. Consequently, the significant increase in PCE for samples A and B can be attributed to their notably higher Jsc.

In photovoltaic devices, the electron-hole pairs are generated through photon absorption. Photocurrent is only generated when electrons and holes are separated by the built-in electric field, and then drift toward the N-region and P-region, respectively. However, if electrons and holes are recombined during this process, the collection efficiency of photogenerated carriers will be reduced, resulting in a decrease in PCE [31]. In short, the recombination process of photogenerated carriers in the I-region deteriorates the photovoltaic performance of solar cells.

**Figure 2:**
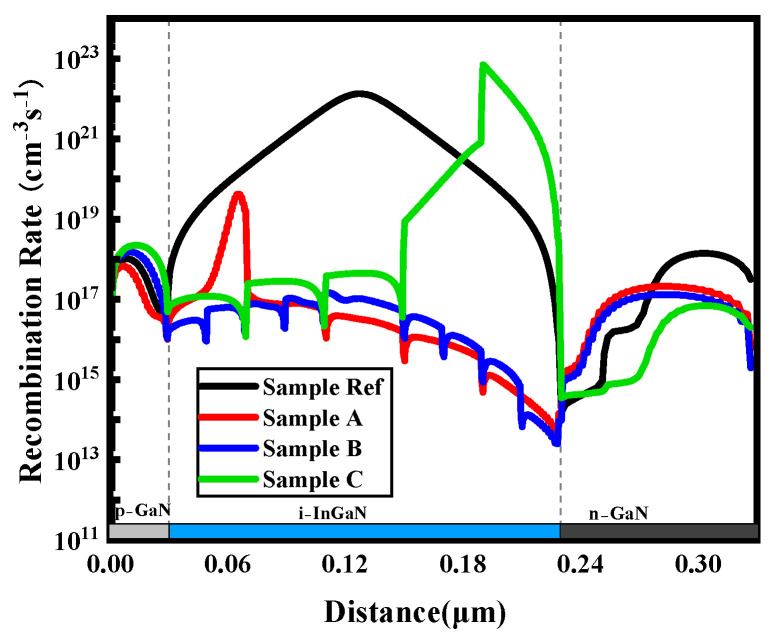
Distribution of carrier recombination rates within four groups of samples.

Therefore, the carrier recombination rates of the four samples are simulated and depicted in Figure 2. Compared with samples A, B, and C, the carrier recombination rate of sample Ref is overall higher, with the maximum recombination rate occurring in the middle of the I-region. For sample A, the In content in the I-region exhibits a step-gradient distribution, decreasing from 50% at the top to 10% at the bottom. The absorption of longer-wavelength photons is enabled in the upper portion of the I-region because the bandgap is narrowed by the higher In content. Thus, the upper portion of the I-region absorbs more photons, generating a higher concentration of photogenerated electrons and holes. According to Equation (2) in the SRH recombination model, the recombination rate (R) is proportional to the product of electron and hole concentrations (*R* ∝ *n·p*). Consequently, the enhanced carrier recombination rate in the upper portion of the I-region results from the increased concentration of photogenerated carriers. As a result, the peak of the carrier recombination rate curve is located near the top of the I-region. Conversely, for sample C, the In content in the I-region exhibits a step-gradient distribution, increasing from 10% at the top to 50% at the bottom. Therefore, the peak of the carrier recombination rate is located near the bottom of the I-region. In contrast to samples A and C, in general, the carrier recombination rate curve of sample B is the lowest and relatively uniform within the entire I-region. Notably, the carrier recombination rate near the P-region is higher than that near the N-region, although the distribution of In content is the same in both the upper and lower portions of the I-region. The reason is that for the front-illuminated photovoltaic devices, the photons absorbed in the lower portion of the I-region are limited due to the absorption in the upper portion. Therefore, the concentration of photogenerated carriers is diminished in the lower portion of the I-region, which leads to a lower carrier recombination rate.

Furthermore, considering the overall recombination process within the I-region, the integrated recombination rates for samples Ref, A, B, and C are 6.7 × 10^22^ s^−1^, 4.4 × 10^19^ s^−1^, 1.65 × 10^18^ s^−1^, and 1.35 × 10^22^ s^−1^, respectively. Remarkably, the integrated carrier recombination rate of sample Ref is the highest, indicating severe carrier recombination loss. Consequently, the collection efficiency of photogenerated carriers is markedly reduced, resulting in degraded photovoltaic performance, which is consistent with the results in Table 1. In contrast, the integrated recombination rates of samples A, B, and C are lower. It means that carrier recombination is suppressed in InGaN solar cells with a step-gradient distribution of In content.

Interestingly, the integrated carrier recombination rate of sample A is approximately 27 times higher than that of sample B, which implies significant carrier recombination in sample A. However, as shown in Table 1, the Jsc of sample A is 7.8% higher than that of sample B. In other words, the higher integrated carrier recombination rate of sample A observed in Figure 2 contradicts its larger Jsc in Table 1.

**Figure 3:**
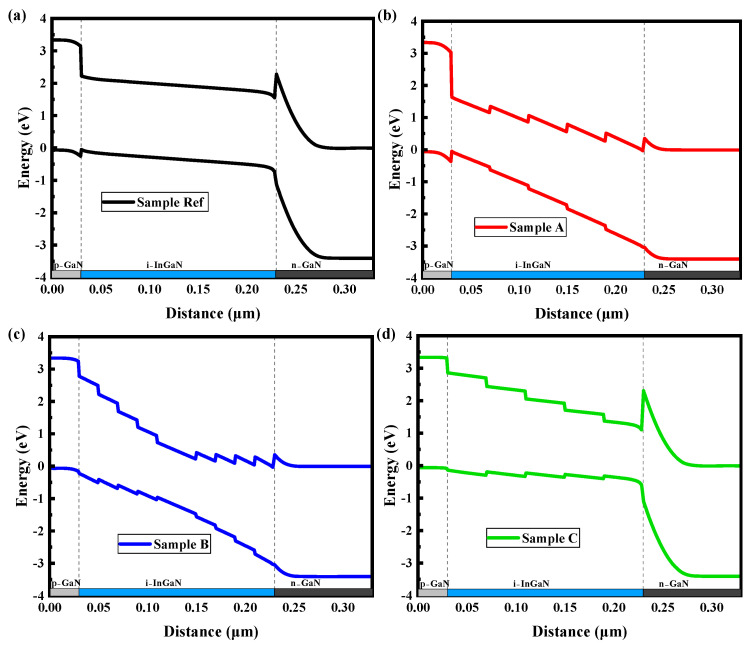
Energy band diagrams of samples (**a**) Ref, (**b**) A, (**c**) B, and (**d**) C.

To explain the previously observed contradiction, it is crucial to consider the significant impact of the energy barrier at the heterojunction interface on carrier transport properties [21]. After comprehensively considering the combined effects of the electric field, the distribution of the In content, polarization effect, and other models, the energy band structures of four groups of samples are shown in Figure 3. As illustrated in Figure 3a,d, the energy band of samples Ref and C are similar, i.e., a high energy barrier is exhibited at the I-region/N-region interface for both samples. It is known that electrons are drifted towards the N-region by the built-in electric field, while a high energy barrier impedes the movement of electrons and leads to electron accumulation at the interface, thereby increasing carrier recombination. As a result, the transport of electrons to the N-region is blocked, diminishing the generation of photocurrent. As shown in Figure 3b,c, the energy barrier height at the I-region/N-region interface is significantly reduced in the energy bands of samples A and B, which can enhance the electron transport and ultimately improve the Jsc and PCE of the device [24]. The above discussion is consistent with the integral carrier recombination in Figure 2 and performance parameters in Table 1. Additionally, it is observed that the step-gradient distribution of In content results in a step-like energy distribution in the conduction band/valence band, which can facilitate the directed drift of carriers. The holes drifting to the P-region in sample A and the electrons drifting to the N-region in sample C are both accelerated along the steps, which reduces the carrier concentration at the interfaces of different In contents. Sample B is slightly different in that electron drift is accelerated in the first half of the I-region and hole drift is accelerated in the second half, resulting in a decrease in the carrier concentration at the interfaces of different In contents. According to Equation (2), the carrier recombination rate is proportional to the carrier concentration. Consequently, it can be seen in Figure 2 that the carrier recombination rate at the step-gradient interface of In content is decreased.

Notably, in Figure 3c, a more pronounced tilt of the conduction band in the upper portion of the I-region is displayed in sample B compared with sample A, implying that the drift of electrons can be facilitated. Thus, a higher photocurrent is expected for sample B than for sample A. However, the Jsc of sample B is lower than that of sample A in Table 1, indicating that other factors must be considered and investigated.

The above analysis indicates that carrier transport significantly affects device performance. To further investigate the effect of reduced energy barrier height on carrier transport process and device performance, sample New is designed by introducing a 40-nm-thick InGaN layer with 10% In content at the I-region/N-region interface, using sample Ref as the reference structure. The performance parameters of samples New, Ref, and A are presented in Table 2 for clear comparison. The results indicate that, compared with sample Ref, the Jsc of sample New is higher by 7.53 mA/cm^2^, ultimately resulting in an 8.22% higher PCE. However, compared with sample A, the Jsc of sample New is lower by 2.77 mA/cm^2^, ultimately leading to a 1.99% lower PCE. To avoid data complexity and analysis difficulties caused by excessive samples, the detailed analysis is provided in the Supplementary Information.

**Figure 4:**
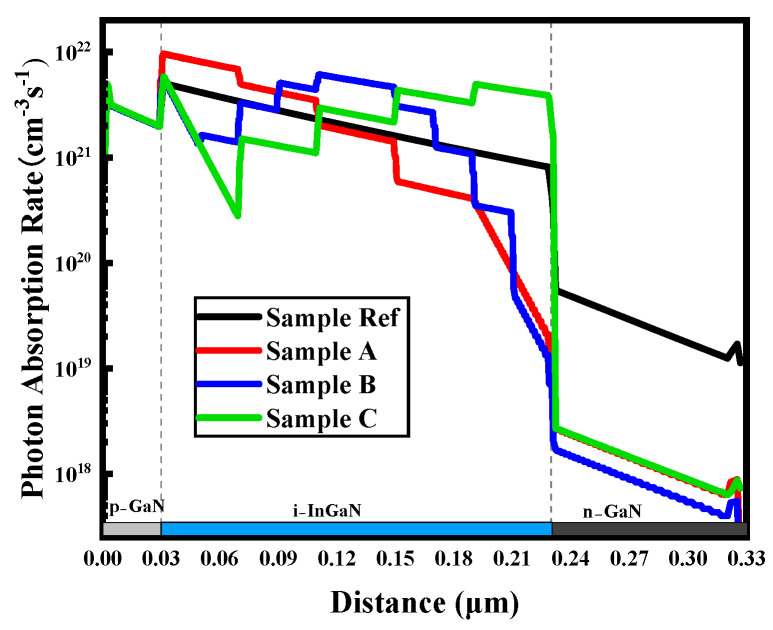
Photon absorption rate of the four groups of samples.

The curves of photon absorption rates for all samples are extracted and shown in Figure 4. According to the basic principle of light absorption, as incident light penetrates deeper into the I-region, its intensity is decreased. Therefore, the light intensity is decreased along the direction of incidence within the I-region, resulting in a decreased photon absorption [26]. Accordingly, the photon absorption rate of sample Ref is gradually decreased along the I-region in Figure 4. For sample A, the highest absorption rate is observed at the upper portion of the I-region due to the 50% In content, which enables the absorption of a wider range of the solar spectrum. As the In content is decreased in a step-gradient distribution to 10% at the bottom of the I-region, the photo absorption rate is decreased. As a result, the photon absorption rate of sample A within the I-region is gradually decreased in a stepwise shape from top to bottom in Figure 4. In contrast, for sample C, the In content is gradually increased in a step-gradient distribution from 10% at the top to 50% at the bottom. Consequently, the photon absorption rate curve of sample C within the I-region gradually increased in a stepwise shape from the top to the bottom in Figure 4. For sample B, the In content in the I-region is increased in a step-gradient distribution from 10% at the top to 50% in the middle and then is decreased to 10% at the bottom. As a result, the photon absorption rate of sample B is increased in a step-gradient distribution and then is decreased, with the highest photon absorption rate appearing in the middle of the I-region.

Furthermore, the integrated photon absorption rates for samples Ref, A, B, and C are 5.30 × 10^22^ s^−1^, 6.65 × 10^22^ s^−1^, 6.34 × 10^22^ s^−1^, and 6.29 × 10^22^ s^−1^, respectively. Remarkably, the integrated photon absorption rate of sample Ref is the lowest due to its uniform In content of 30%, which limits the absorption of photons with energies above 2.296 eV. In contrast, the integrated photon absorption rates of samples A, B, and C are higher due to the maximum In content of 50% in the I-region, which allows for the absorption of a wider range of the solar spectrum and consequently results in a greater number of absorbed photons. Among the samples with a step-gradient distribution of In content, the integrated photon absorption rate of sample C is the lowest. As mentioned previously, for front-illuminated photovoltaic devices, since the incident photons are absorbed by the upper and middle portions of the I-region, a limited number of photons can be absorbed at the bottom of the I-region. As a result, although the In content is higher at the bottom of the I-region, the limited number of available photons leads to a lower integrated photon absorption rate for sample C. In contrast, the high In content regions of samples A and B are located at the upper portion of the I-region, which is beneficial to photo absorption, and thus the integrated photon absorption rates are larger. Notably, the integrated photon absorption rate of sample A is higher than that of sample B. Similar to the discussion about sample C, although the In content at the middle of the I-region in sample B is the highest, the limited number of available photons leads to a lower integrated photon absorption rate for sample B.

Therefore, the integrated photon absorption rate of sample A is higher than that of sample B. Coupled with the lower carrier recombination rate and efficient carrier transport in sample A, the Jsc and PCE of sample A are the highest among all samples, which is consistent with the results presented in Table 1.

## 4. Discussion

In summary, the photovoltaic performance of four P-I-N type InGaN solar cells with different distributions of In content within the active region is numerically investigated and compared. According to our study, compared with the traditional P-I-N structure with uniformly distributed In content, the photovoltaic performance of the sample with a step-gradient distribution of the In content in the I-region can be enhanced. Specifically, for sample A with In content in the I-region step-gradient decreases from 50% at the top to 10%, the PCE is increased from 0.08% to 10.29%. This improvement is attributed to the Jsc being increased from 0.05 mA/cm^2^ to 10.35 mA/cm^2^, which is attributed to several factors, including the reduction of carrier recombination, an energy band structure that facilitates efficient carrier transport, and, most importantly, the enhanced photon absorption. Additionally, sample New is designed based on sample Ref, demonstrating that the carrier transport process of the device is effectively facilitated by the introduction of a 10-In-content InGaN layer in the I-region, resulting in enhanced performance. It is suggested that the distribution of In content in the I-region is rationally designed to enhance the PCE from the device structural design aspect, providing theoretical foundations and conceptual guidance for the development of high-performance P-I-N type InGaN solar cells.

## Figures and Tables

**Table 1:** Comparison of photovoltaic performance of four groups of samples.

Sample (10 × 10 μm^2^)	V_OC_ (V)	J_SC_ (mA/cm^2^)	FF (%)	PCE (%)
Sample Ref	1.79	0.05	81.57	0.08
Sample A	1.16	10.35	85.64	10.29
Sample B	1.14	9.60	83.37	9.18
Sample C	1.82	0.06	90.06	0.10

**Table 2:** Device performance parameters for sample Ref, sample A, and sample New.

Sample	V_OC_ (V)	J_SC_ (mA/cm^2^)	FF (%)	PCE (%)
Sample New	1.40	7.58	77.89	8.30
Sample Ref	1.79	0.05	81.57	0.08
Sample A	1.16	10.35	85.64	10.29

## Data Availability

The data presented in this study are available on reasonable request from the corresponding author.

## Supplementary Materials

The following supporting information can be downloaded at: https://www.mdpi.com/article/10.3390/mi16070833/s1, Figure S1: (a) Schematic diagram of Sample New; (b) J-V characteristic curves of Sample Ref, Sample A, and Sample New. Figure S2: Energy band diagram of (a) Sample New; (b) Sample Ref; (c) Sample A; (d) Photon absorption rates of Sample Ref, Sample A, and Sample New. Table S1: Material parameters in the simulation model. Table S2: Device Performance Parameters for Sample Ref, Sample A, and Sample New.
