# Supplementary material for: Investigation on the Carrier Dynamics in P-I-N Type Photovoltaic Devices with Different Step-Gradient Distribution of Indium Content in the Intrinsic Region
Source: Micromachines (Basel). 2025 Jul 21;16(7):833. doi: 10.3390/mi16070833 (PMC12298683; doi:10.3390/mi16070833)
Supplement: Supplementary file 1 [file micromachines-16-00833-s001.zip › micromachines-3737022-supplementary.pdf]

micromachines

MDPI

Supplementary Information: Investigation on the Carrier Dynamics in P-I-N Type Photovoltaic Devices with Different Step-Gradient Distribution of Indium Content in the Intrinsic Region

Yifan Song, Wei Liu *, Junjie Gao, Di Wang, Chengrui Yan, Bohan Shi, Linyuan Zhang, Xinnan Zhao and Zeyu Liu

School of Microelectronics, Northwestern Polytechnical University, Xi'an 710072, China; syf1274639733@mail.nwpu.edu.cn (Y.S.); gaojunjie1030@mail.nwpu.edu.cn (J.G.); wangdi3043@mail.nwpu.edu.cn (D.W.); 15251751098@mail.nwpu.edu.cn (C.Y.); bohanshi@mail.nwpu.edu.cn (B.S.); 2023303024@mail.nwpu.edu.cn (L.Z.); zhaoxinnan@mail.nwpu.edu.cn (X.Z.); 2021264377@mail.nwpu.edu.cn (Z.L.)

* Correspondence: liuwei127@nwpu.edu.cn

1. The Specific Values of the Parameters Used in the Simulation

Table S1. Material parameters in the simulation model.

Parameters	GaN	InN	$In_{0.1}Ga_{0.9}N$	$In_{0.2}Ga_{0.8}N$	$In_{0.3}Ga_{0.7}N$	$In_{0.4}Ga_{0.6}N$	$In_{0.5}Ga_{0.5}N$
E_g(eV)	3.4	0.7	3.01	2.63	2.28	1.98	1.69
Electron mass m_e/m_0	0.2	0.05	0.185	0.170	0.155	0.140	0.125
Hole mass m_p/m_0	1.25	0.6	1.185	1.150	1.115	1.080	1.045
Affinity (eV)	4.1	5.9	4.373	4.639	4.884	5.094	5.297
Permittivity $\varepsilon_r/\varepsilon_0$	8.9	10.5	9.06	9.22	9.38	9.54	9.70
$\mu_{min,e}$ ($cm^2V^{-1}S^{-1}$)	55	30	Linear interpolation*	Linear interpolation*	Linear interpolation*	Linear interpolation*	Linear interpolation*
$\mu_{max,e}$ ($cm^2V^{-1}S^{-1}$)	1000	1100	Linear interpolation*	Linear interpolation	Linear interpolation	Linear interpolation*	Linear interpolation*
$\mu_{min,h}$ ($cm^2V^{-1}S^{-1}$)	3	3	Linear interpolation*	Linear interpolation*	Linear interpolation*	Linear interpolation*	Linear interpolation*
$\mu_{max,h}$ ($cm^2V^{-1}S^{-1}$)	170	340	Linear interpolation*	Linear interpolation*	Linear interpolation*	Linear interpolation*	Linear interpolation*
r_e	0.95	0.7	Linear interpolation*	Linear interpolation*	Linear interpolation*	Linear interpolation*	Linear interpolation*
r_h	2	2	Linear interpolation*	Linear interpolation*	Linear interpolation*	Linear interpolation*	Linear interpolation*
$N_{g,e}$ (cm^{-3})	1.8×10^{17}	2×10^{16}	Linear interpolation*	Linear interpolation*	Linear interpolation*	Linear interpolation*	Linear interpolation*
$N_{g,h}$ (cm^{-3})	3×10^{17}	1.8×10^{17}	Linear interpolation*	Linear interpolation*	Linear interpolation*	Linear interpolation*	Linear interpolation*
C_{13} (Gpa)	106	92	——	——	——	——	——
C_{33} (Gpa)	398	224	——	——	——	——	——
E_{31} (C/m^2)	-0.49	-0.57	——	——	——	——	——
E_{33} (C/m^2)	0.73	0.97	——	——	——	——	——
P_{sp} (C/m^2)	-0.029	-0.032	——	——	——	——	——

*Note: Linear interpolation is a mathematical method for estimating intermediate values based on known data points. In InxGa1-xN materials, linear interpolation is used to calculate material parameters at different In contents based on the material parameter values of GaN and InN.

2. Structural and Performance Analysis of the Sample New

We designed Sample New by introducing a 40 nm thick InGaN layer with 10% indium (In) content at the I-region/N-region interface, using Sample Ref as the reference structure. The device structure of Sample New is illustrated in Figure S1(a).

Table S2. Device Performance Parameters for Sample Ref, Sample A, and Sample New.

Sample	V_{OC} (V)	J_{SC} (mA/cm^2)	FF (%)	PCE (%)	Integral value of photon absorption rate (s^{-1})
Sample New	1.40	7.58	77.89	8.30	4.89×10^{22}
Sample Ref	1.79	0.05	81.57	0.08	5.30×10^{22}
Sample A	1.16	10.35	85.64	10.29	6.65×10^{22}

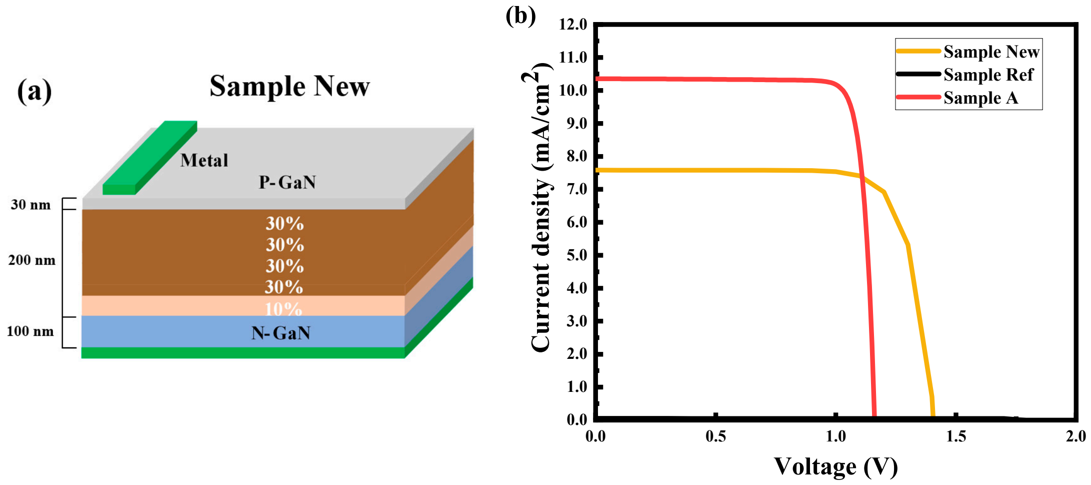

Figure S1. (a) Schematic diagram of Sample New; (b) J-V characteristic curves of Sample Ref, Sample A, and Sample New.

The J-V characteristic curves of Sample New, Sample Ref, and Sample A are shown in Figure S1(b), with specific parameters listed in Table S2. The results demonstrate that compared to Sample Ref, Sample New exhibits a significant increase in short-circuit current density (Jsc) from 0.05 mA/cm^2 to 7.58 mA/cm^2, and consequently, the power conversion efficiency (PCE) improves from 0.08% to 8.3%. To further analyze the increase of Jsc for Sample New, its energy band diagram is simulated, as shown in Figure S2(a). Due to the insertion of a 10%-In-content InGaN layer, energy band bending occurs near the N-region, and the energy barrier height between the N-region and I-region is 0.43 eV. Compared with the energy band diagram of Sample Ref in Figure S2(b), the 10%-In-content InGaN layer in Sample New effectively reduces the energy barrier height between the I-region and N-region from 0.84 eV to 0.43 eV. It facilitates the carrier transport process, resulting in an increase of 7.53 mA/cm^2 in Jsc and ultimately an enhancement of 8.22% in PCE for Sample New.

On the other hand, as shown in Figure S1(b) and Table S2, although the PCE of Sample New is 8.22% higher than that of Sample Ref, it is still 1.99% lower than that of Sample A, primarily due to that the Jsc of 7.58 mA/cm^2 of Sample New is lower than that of Sample A, which is 10.35 mA/cm^2. Detailed comparative analysis of the energy band diagrams in Figures S2(a) and (c) reveals that both Sample New and Sample A exhibit essentially identical energy barrier heights between the I-region and N-region (i.e. 0.43 eV and 0.44 eV, respectively), since the In content in InGaN layers near the N-region is identical for both Samples. Therefore, compared to Sample A, the lower Jsc of Sample New cannot be

attributed to the energy barrier height at the I-region/N-region interface. Furthermore, the photon absorption rates of Sample New and Sample A are simulated, as shown in Figure S2(d), with specific parameters listed in Table S2. The integrated value of photon absorption rates for Sample New and Sample A are 4.89×10^{22} s^{-1} and 6.65×10^{22} s^{-1}, respectively, with the integrated value of photon absorption rate of Sample New being 1.76×10^{22} s^{-1} lower than that of Sample A. As mentioned in our article, enhancing photon absorption can increase the Jsc of the device. Consequently, the reduced integrated value of photon absorption rate for Sample New results in a 2.77 mA/cm² lower Jsc compared to Sample A. Therefore, the PCE of Sample New is 1.99% lower than that of Sample A.

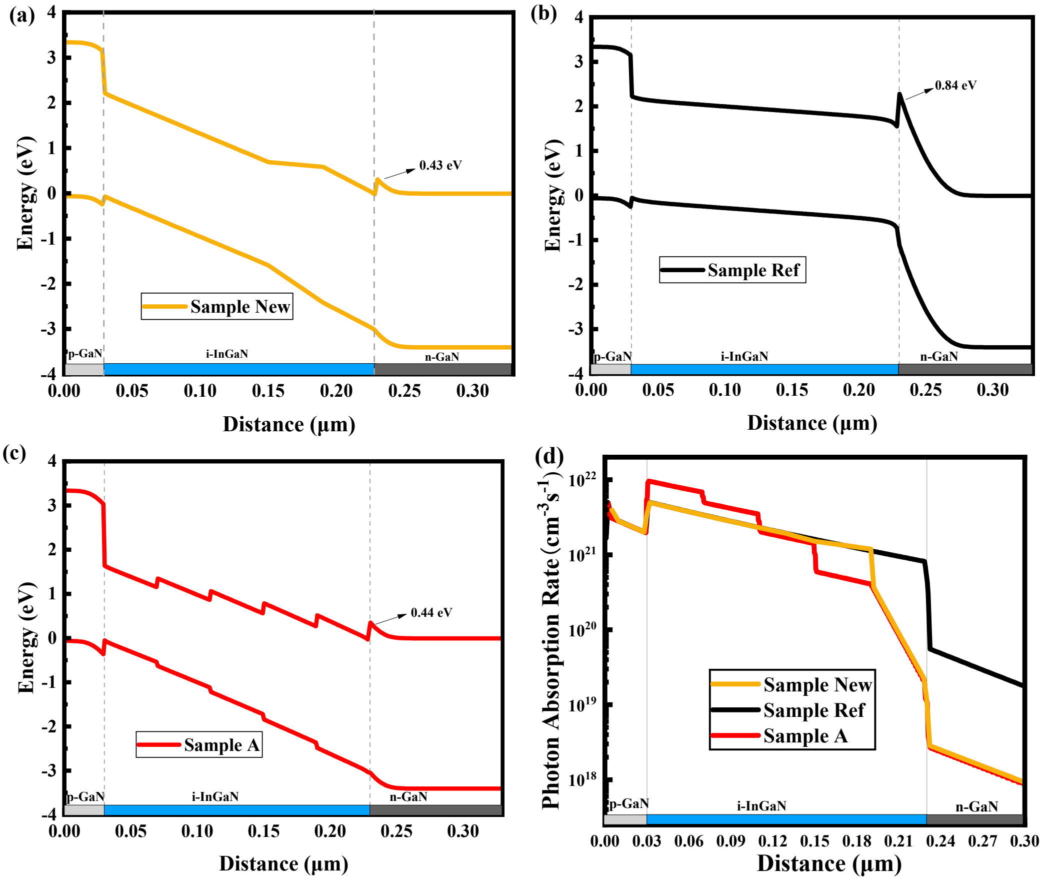

Figure S2. Energy band diagram of (a) Sample New; (b) Sample Ref; (c) Sample A; (d) Photon absorption rates of Sample Ref, Sample A, and Sample New.
